# Immunological Characteristics of Hepatic Dendritic Cells in Patients and Mouse Model with Liver *Echinococcus multilocularis* Infection

**DOI:** 10.3390/tropicalmed9050095

**Published:** 2024-04-25

**Authors:** Hui Wang, Yinshi Li, Qian Yu, Mingkun Wang, Abidan Ainiwaer, Na Tang, Xuran Zheng, Adilai Duolikun, Bingqing Deng, Jing Li, Yujuan Shen, Chuanshan Zhang

**Affiliations:** 1 State Key Laboratory of Pathogenesis, Prevention and Treatment of High Incidence Diseases in Central Asia, Clinical Medicine Institute, The First Affiliated Hospital of Xinjiang Medical University, Urumqi 830054, China; 2 Basic Medical College, Xinjiang Medical University, Urumqi 830011, China; 3 National Institute of Parasitic Diseases, Chinese Center for Disease Control and Prevention (Chinese Center for Tropical Diseases Research), NHC Key Laboratory of Parasite and Vector Biology (National Institute of Parasitic Diseases, Chinese Center for Disease Control and Prevention), World Health Organization Collaborating Centre for Tropical Disease, Shanghai 200025, China

**Keywords:** alveolar echinococcosis, liver, dendritic cells, costimulatory molecules, immune checkpoint molecules, ligands

## Abstract

The cestode *Echinococcus multilocularis,* which mainly dwells in the liver, leads to a serious parasitic liver disease called alveolar echinococcosis (AE). Despite the increased attention drawn to the immunosuppressive microenvironment formed by hepatic AE tissue, the immunological characteristics of hepatic dendritic cells (DCs) in the AE liver microenvironment have not been fully elucidated. Here, we profiled the immunophenotypic characteristics of hepatic DC subsets in both clinical AE patients and a mouse model. Single-cell RNA sequencing (scRNA-Seq) analysis of four AE patient specimens revealed that greater DC numbers were present within perilesional liver tissues and that the distributions of cDC and pDC subsets in the liver and periphery were different. cDCs highly expressed the costimulatory molecule CD86, the immune checkpoint molecule CD244, LAG3, CTLA4, and the checkpoint ligand CD48, while pDCs expressed these genes at low frequencies. Flow cytometric analysis of hepatic DC subsets in an *E. multilocularis* infection mouse model demonstrated that the number of cDCs significantly increased after parasite infection, and a tolerogenic phenotype characterized by a decrease in CD40 and CD80 expression levels was observed at an early stage, whereas an activated phenotype characterized by an increase in CD86 expression levels was observed at a late stage. Moreover, the expression profiles of major immune checkpoint molecules (CD244 and LAG3) and ligands (CD48) on hepatic DC subsets in a mouse model exhibited the same pattern as those in AE patients. Notably, the cDC and pDC subsets in the *E. multilocularis* infection group exhibited higher expression levels of PD-L1 and CD155 than those in the control group, suggesting the potential of these subsets to impair T cell function. These findings may provide valuable information for investigating the role of hepatic DC subsets in the AE microenvironment and guiding DC targeting treatments for AE.

## 1. Introduction

Alveolar echinococcosis (AE), like a slow-growing malignant tumor, is a lethal parasitic disease caused by the continuous infiltrative growth of *Echinococcus multilocularis* (*E. multilocularis*) larvae (metacestode) [1]. The larvae reside predominantly in liver organ and can spread to lung or brain organs, seriously threatening human health [2]. The World Health Organization (WHO) reported that the 10 year mortality rate will reach 90% if patients are left untreated [3,4]. Despite the many surgical therapeutics available for treating hepatic AE patients, the risk of recurrence after surgical treatment remains the main problem in the treatment of this disease, and 30% of patients are still not eligible for surgical treatment [5]. Therefore, elucidating the parasite immune evasion mechanisms is necessary for exploring effective treatment strategies to improve the survival of patients with AE.

The outcome of AE development depends on the immune status of the host, and rapid and unlimited parasite proliferation occurs in humans coinfected with *E. multilocularis* and the human immunodeficiency virus (HIV) [6,7]. In recent years, accumulating literature has focused on investigating T cell roles in AE pathogenesis, and impaired T cell responses have been reported, leading to liver immunosuppressive microenvironment formation and contributing to *E*. *multilocularis* chronic infection [8,9,10,11]. Mechanistically, infiltrating T cells in the hepatic AE lesion microenvironment exhibit high and sustained expression of immune checkpoint receptors, such as lymphocyte activation gene-3 (LAG3), programmed cell death-1 (PD-1), T cell immunoreceptor with Ig and ITIM domains (TIGIT), and 2B4 (CD244), which mediate *E. multilocularis* immune escape by impairing T cell functions [9,12]. Dendritic cells (DCs) are highly heterogeneous cell populations, and different DC populations exhibit different phenotypes and functions [13]. It has been reported that DCs play a critical role in the Th1 to Th2 shift in establishing immune evasion during the chronic phase of AE [8,14,15]. However, the phenotypic characteristics and functional diversity of the hepatic DC subsets in the *E*. *multilocularis*-infected liver microenvironment have not been fully investigated.

Generally, DCs include conventional DCs (cDCs) and plasmacytoid DCs (pDCs) [16,17,18]. As the most important antigen-presenting cells (APCs), cDCs have an intrinsic ability to efficiently take up and cross-present antigens to activate T cell responses against intracellular and extracellular pathogens infection [19]. In contrast to cDCs, pDCs are relatively poor APCs and can rapidly produce large amounts of type I interferon (IFN I) in response to pathogen infection [20]. The maturation/activation status of DCs determines their impact on adaptive immune responses. Fully activated DCs are characterized by high expressions of CD80, CD86, and CD40 and induce T cell responses, whereas inactivated DCs are characterized by low expressions of CD80, CD86, and CD40 and induce T cell tolerance [21]. Moreover, it has been reported that some inhibitory checkpoint molecules, such as LAG3 [22] or their ligands (PD-L1/2), expressed on the DC [23] can lead to tolerogenic DCs. Although previous studies have reported that the excreted/secreted (E/S) products of *E. multilocularis* larvae do not induce DC maturation in vitro [24] and that the expression of DC maturation markers on peritoneal DCs isolated from an intraperitoneal *E. multilocularis* infection mouse model is downregulated [8], little is known about the maturation status of hepatic cDC and pDC subsets and the expression level of inhibitory checkpoint molecules or their ligands on their surface during persistent infection.

In our present study, we aim to investigate the expression profiles of costimulatory molecules, immune checkpoint molecules, and their ligands on hepatic DC subsets in both AE patients and *E. multilocularis* infection mice models.

## 2. Materials and Methods

### 2.1. Human Subjects

AE patients were diagnosed and accepted surgical treatment at the First Affiliated Hospital of Xinjiang Medical University, Urumqi, China. The perilesion (PL) and adjacent normal (AN) liver tissue samples were collected and treated according to our previous study [9]. All of the human participants enrolled in this study were treated in accordance with the Declaration of Helsinki and approved by the Ethics Committee of the First Affiliated Hospital of Xinjiang Medical University (Approval No. K202206-27), and all of the participants provided signed informed consent.

### 2.2. Mice

C57BL/6 mice (eight to ten weeks, female) were purchased from Beijing Vital River Experimental Animal Technology Co., Ltd. (Beijing, China) and housed under specific pathogen-free conditions at the Animal Facility of Xinjiang Medical University. All mice received humane care, and all the procedures and experiments were approved by the Experimental Animal Ethics Committee of Xinjiang Medical University (No: IACUC-20210315-01).

### 2.3. Hepatic Mouse Model of E. multilocularis Infection

The hepatic experimental *E. multilocularis* infection mouse model was established according to our previous methods [11]. Briefly, *E. multilocularis* protoscoleces (PSCs) were isolated from the infected gerbils under a sterile environment, as described in our previous study [25]. A total of 20 mice were randomly allocated into 2 groups of 10 mice each. Mice in the *E. multilocularis* infection group were infected with 2000 live PSCs in RPMI 1640 medium (Gibco, Auckland, New Zealand) via the hepatic portal vein, while mice in the control group (Con) were injected with the same volume of RPMI 1640 alone. At 2 or 24 weeks after infection, 4–6 mice per group were anesthetized and sacrificed. The whole livers from each group mice were collected for flow cytometry analysis.

### 2.4. Flow Cytometric Analysis

The nonparenchymal liver cells (NPLCs) were isolated from the whole liver of each mouse as previously described [26] to analyze the hepatic DC subset composition and the major activating and inhibitory molecules or ligands expression on hepatic DCs at 2 and 24 weeks. Briefly, mice were anesthetized and fixed on a dissecting board, and a laparotomy was performed to expose the liver and the portal vein, followed by perfusion of the liver with PBS using a 0.45 × 15RWLB venous infusion needle (Zhejiang Kindly Medical Devices Co., Ltd, Wenzhou, China). Once the liver became pale, we immediately removed the liver, which was placed into a sterile 6 mm dish, homogenized with PBS containing 0.2% bovine serum albumin (BSA), passed through a steel mesh, and resuspended. Then, the NPLCs were isolated by centrifugation with 40% Percoll (GE Healthcare, Little Chalfont, UK) for 20 min; red blood cells were depleted using red blood lysis buffer (Biolegend, San Diego, CA, USA). The cells were then counted on a hemocytometer in the presence of trypan blue. The 1.0 × 10^6^ NPLCs were washed and incubated in PBS containing 2% BSA and 0.1% sodium azide for 20 min at 4 °C with an anti-mouse CD16/CD32 monoclonal antibody (mAb) to block any nonspecific antibody binding. Then, the cells were stained with a mixture of surface antibodies for 30 min at 4 °C in the dark. The antibodies used in this study are listed in Table S1. The cells were evaluated on an LSRFortessa flow cytometer (BD Immunocytometry Systems, San Jose, CA, USA), and the data were analyzed using FlowJo software (version V10; Tree Star, Inc., Ashland, OR, USA).

### 2.5. Statistical Analysis

For data analysis, we used the GraphPad Prism software (version 8, GraphPad, CS, USA), employing a paired t-test to compare the percentage of cells with positive CD11c staining in the paired AN and PL tissues of AE patients and an independent sample t-test to compare the expression (percentages, absolute numbers, or MFI) of costimulatory molecules, immune checkpoint molecules, and their ligands on hepatic DC subsets between the control group and the *E. multilocularis* infection group. Data are presented as the mean ± standard error of the mean (SEM). A *p* value < 0.05 was considered to indicate statistical significance.

## 3. Results

### 3.1. scRNA-seq Reveals the Distribution and Molecular Expression of DC Subsets in the Hepatic AE Tissue Microenvironment

**Figure 1:**
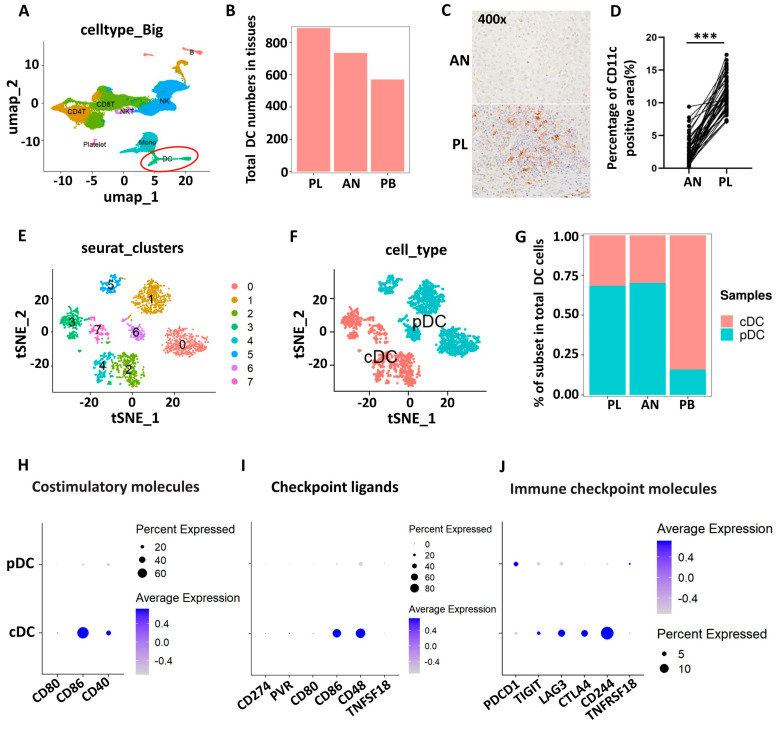
Characterization of DC subset single-cell landscapes in AE patients. (**A**) UMAP plot representing the 8 large cell type clusters from 4 AE patients. (**B**) The number of total DCs in PL, AN, and PB specimens. (**C**) Representative immunohistochemical staining for CD11c in paired liver tissue samples (AN versus PL) from 42 AE patients (400× indicates magnification). (**D**) The percentage of cells with positive CD11c staining was quantified using cellSens Dimension software (version 1.9). (**E**) TSNE plots representing DC clusters (n = 8). (**F**) The 8 DC clusters were divided into two main DC subsets (cDCs and pDCs) according to characteristic genes. (**G**) The percentages of total DC and pDC subsets in different tissues. (**H**–**J**) The expression of costimulatory molecules, immune checkpoint ligands, and immune checkpoint molecules on cDC and pDC subsets. PL: perilesional liver tissue approximately 0.5 cm from the metacestode lesion; AN: adjacent normal liver tissue at least 2 cm from the lesion. *** *p* < 0.001.

A 10 × 5′ single-cell sequencing (scRNA-Seq) dataset of CD45^+^ immune cells isolated from PB, PL, and AN liver tissue samples from four AE patients (accession no. HRA000553; https://figshare.com/s/f2a4578be27c2cb767f9, accessed on 16 April 2022) [27]) was first analyzed. As illustrated in the uniform manifold approximation and projection (UMAP) plots shown in Figure 1A, CD45^+^ cells were divided into eight major clusters using an unsupervised clustering method; these included CD4^+^ T cells, CD8^+^ T cells, natural killer T (NKT) cells, NK cells, B cells, monocytes, dendritic cells (DCs), and platelets. We obtained a total of 888 DCs from the PL samples, which was greater than those from the AN and PB samples (734 and 570) (Figure 1B). Immunohistochemical staining confirmed that there were more CD11c^+^ DCs infiltrating the periparasitic area in the PL than in the AN (Figure 1C,D). Next, we reclustered all the DCs and obtained eight subclusters, which were divided into cDC (cluster 2, 3, 4, 7) and pDC (cluster 0, 1, 5, 6) subsets according to their characteristic genes (cDC, CD11c^+^ MHCII^+^; pDC, LILRA4^+^) (Figure 1E,F) [28,29]. The distributions of cDCs and pDCs in the liver and periphery were different. The pDCs were more common in liver tissues than in the periphery. In contrast, there were more cDCs in the periphery than in the liver (Figure 1G). In addition, dot plot and violin plot analyses revealed that cDCs expressed CD86 and CD40 with high levels as well as the immune checkpoint ligand CD48 and the immune checkpoint molecules CD244, LAG3, and CTLA4, while pDCs expressed these genes infrequently and at low levels (Figure 1H–J and Figure S1).

### 3.2. Changes in the Hepatic DC Composition in Mouse Livers during E. multilocularis Infection

**Figure 2:**
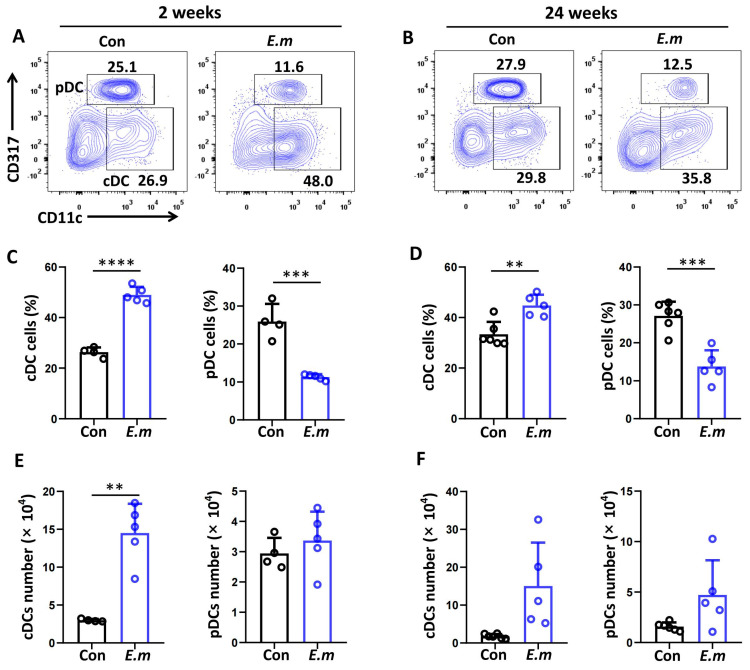
Changes in hepatic DC subsets in mice infected with *E. multilocularis* PSCs during the course of infection. (**A**,**B**) Representative flow cytometry plots of hepatic DC subsets (cDCs: CD11c^hi^CD317^−^; pDCs: CD317^hi^CD11C^int^) in mouse livers infected with the *E. multilocularis* PSC at 2 and 24 weeks. (**C**,**D**) The percentages of cDCs and pDCs in mouse livers infected with *E. multilocularis* PSCs at 2 and 24 weeks. (**E**,**F**) Absolute numbers of cDCs and pDCs in mouse livers infected with *E. multilocularis* PSCs at 2 and 24 weeks. The data are shown as the mean ± SEM (n = 4–6 mice per group). ** *p* < 0.01, *** *p* < 0.001 and **** *p* < 0.0001.

Hepatic DCs can be classified into two main subsets: cDCs (CD45^+^NK1.1^−^CD19^−^CD3^−^MHCII^+^CD11c^hi^CD317^−^) and pDCs (CD45^+^NK1.1^−^CD19^−^CD3^−^MHCII^+^CD317^hi^CD11C^int^) [30]. To assess DC subset changes in whole mouse livers during *E. multilocularis* infection, HNPCs were isolated and analyzed by flow cytometry analysis at 2 and 24 weeks (Figure 2A,B). Compared with the control group, we observed that the proportion of cDCs was significantly increased in the *E. multilocularis* infection group at 2 weeks (Con vs. *E.m* group: 26.30 ± 1.93 vs. 48.96 ± 3.16%, *p* < 0.0001) and 24 weeks (Con vs. *E.m* group: 33.37 ± 4.89 vs. 44.78 ± 4.20%, *p* < 0.01), whereas the proportion of pDCs was significantly decreased in the *E. multilocularis* infection group at 2 weeks (Con vs. *E.m* group: 25.93 ± 4.65 vs. 11.30 ± 0.74%, *p* < 0.001) and 24 weeks (Con vs. *E.m* group: 27.10 ± 3.70 vs. 13.77 ± 4.28%, *p* < 0.001) (Figure 2C,D). The absolute number of cDCs was significantly increased in the *E. multilocularis* infection group at 2 weeks, and the absolute number of pDCs was also increased in the infection group at 2 and 24 weeks but was not significantly different (Figure 2E,F).

### 3.3. Expression of Costimulatory Molecules on Hepatic DC Subsets in Mouse Livers during E. multilocularis Infection

**Figure 3:**
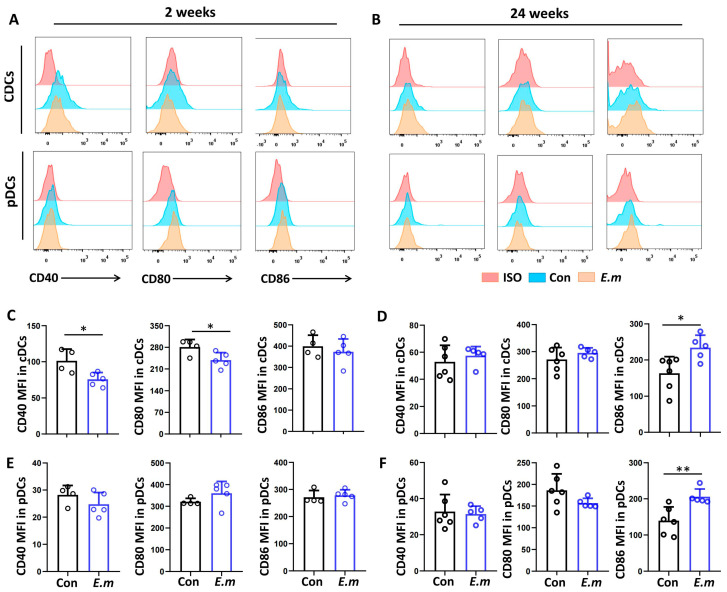
Expression of costimulatory molecules on hepatic DC subsets in *E. multilocularis*-infected mice. (**A**,**B**) Representative histograms of CD40, CD80, and CD86 expressions on hepatic cDCs and pDCs at 2 and 24 weeks after infection. The isotype (shown in red), control (Con, shown in blue), and *E. multilocularis*-infected (*E.m.*, shown in orange) groups were used. (**C**,**D**) MFI of CD40, CD80, and CD86 expressed by cDCs at 2 and 24 weeks after infection. (**E**,**F**) MFI of CD40, CD80, and CD86 expressed by pDCs at 2 and 24 weeks after infection. The data are shown as the mean ± SEM (n = 4–6 mice per group). * *p* < 0.05, ** *p* < 0.01.

As DC activation is necessary for the development and differentiation of effector T cells, the activation or maturation of cDC and pDC subsets in mouse livers during *E. multilocularis* infection was assessed by flow cytometry analysis of the three costimulatory molecules, CD40, CD80, and CD86, and their expressions at 2 and 24 weeks. Compared with the control group, we observed that the expressions of CD40 and CD80, expressed as the MFI, were significantly decreased in the cDC subset in the *E. multilocularis* infection group at 2 weeks, but no change in the pDC population was observed (Figure 3A,C,E). In contrast, the expression of the CD86 was significantly increased in the cDC and pDC subsets in the *E. multilocularis* infection group at 24 weeks after infection (Figure 3B,D,F). These results indicated that hepatic cDC subsets exhibit a tolerogenic phenotype at an early stage, whereas the cDC and pDC subsets exhibit an activated phenotype at a late stage.

### 3.4. Expression of Immune Checkpoint Molecules on Hepatic DC Subsets in E. multilocularis-Infected Mice

**Figure 4:**
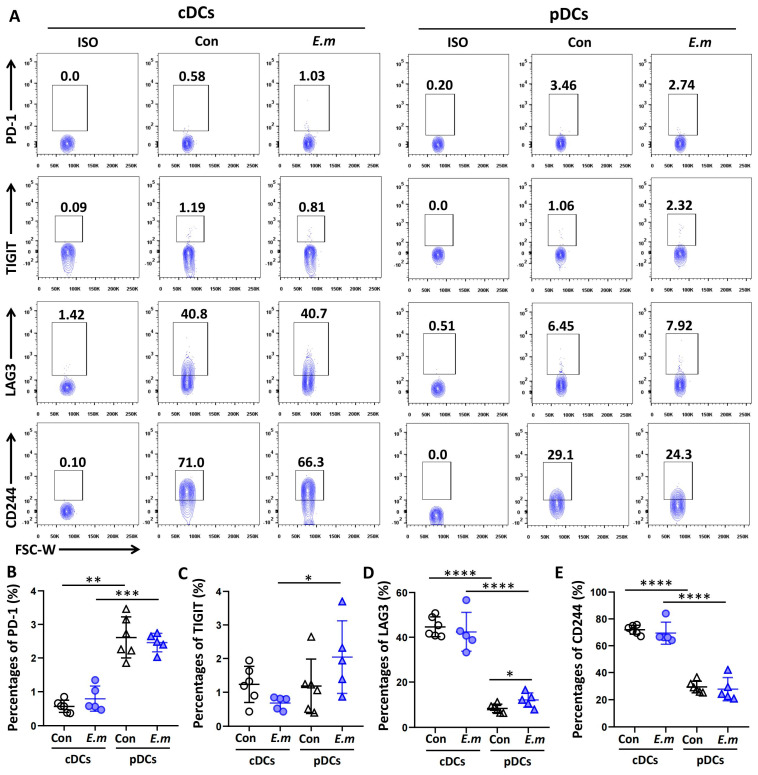
Surface expression of immune checkpoint molecules on hepatic DC subsets in *E. multilocularis*-infected mice. (**A**) Representative flow plots of the immune checkpoint molecules PD-1, TIGIT, LAG3, and CD244 and their expressions in cDCs and pDCs in the livers of mice at 24 weeks after infection. (**B**) The percentages of PD-1^+^cDCs and PD-1^+^pDCs in the livers of the mice at 24 weeks. (**C**) The percentages of TIGIT^+^cDCs and TIGIT^+^pDCs in the livers of mice at 24 weeks. (**D**) The percentages of LAG3^+^cDCs and LAG3^+^pDCs in the livers of mice at 24 weeks. (**E**) The percentages of CD244^+^cDCs and CD244^+^pDCs in the livers of mice at 24 weeks. The data are shown as the mean ± SEM (n = 5–6 mice per group). Circle represents cDCs, and a triangle represents pDCs. * *p* < 0.05, ** *p* < 0.01, *** *p* < 0.001, and **** *p* < 0.0001.

As the expression of immune checkpoint molecules on the DC may contribute to tolerogenic DCs inhibitory properties, we analyzed PD-1, TIGIT, LAG3, and CD244 expressions in cDCs and pDCs in the livers of mice at 24 weeks after infection. As shown in Figure 4, the expressions of these immune checkpoint molecules were quite variable among the cDC and pDC subsets. The expressions of PD-1 and TIGIT were lower in cDCs than in pDCs (Figure 4A–C), whereas the expressions of LAG3 and CD244 were much higher in cDCs than in pDCs (Figure 4A,D,E). Moreover, the expression of CD244 in cDCs was greater than those of LAG3, PD-1, and TIGIT in cDCs, revealing the same expression profile as that observed in AE patients.

### 3.5. Expression of Immune Checkpoint Ligands on Hepatic DC Subsets in E. multilocularis-Infected Mice

**Figure 5:**
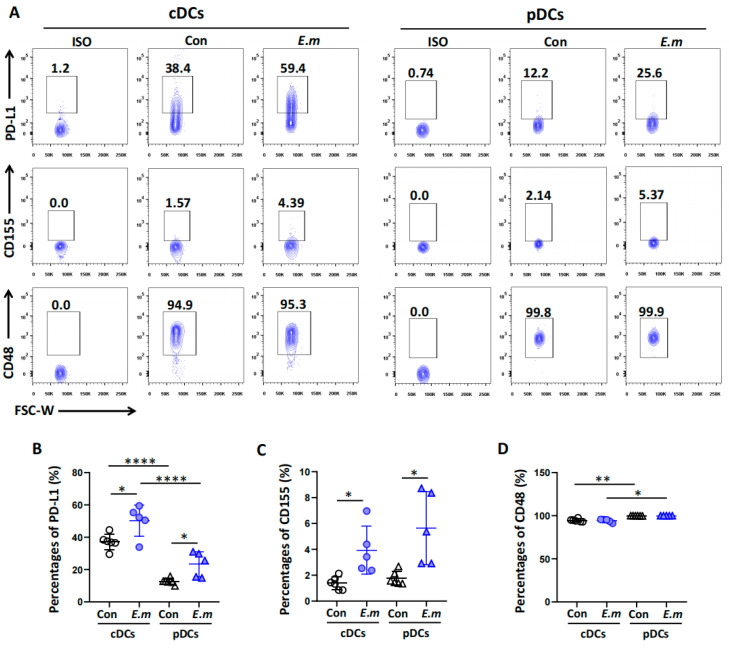
Surface expression of immune checkpoint ligands on hepatic DC subsets in *E. multilocularis*-infected mice. (**A**) Representative flow plots of the expression of the immune checkpoint ligands PD-L1, CD155, and CD48 in cDCs and pDCs in the livers of mice at 24 weeks. (**B**) The percentages of PD-L1^+^ cDCs and PD-L1^+^ pDCs in the livers of the mice at 24 weeks. (**C**) The percentages of CD155^+^cDCs and CD155^+^pDCs in the livers of mice at 24 weeks. (**D**) The percentages of CD48^+^cDCs and CD48^+^pDCs in the livers of mice at 24 weeks. The data are shown as the mean ± SEM (n = 5–6 mice per group). Circle represents cDCs, and a triangle represents pDCs. * *p* < 0.05, ** *p* < 0.01 and **** *p* < 0.0001.

The binding of T cell surface immune checkpoint receptors to adjacent cell surface immune checkpoint ligands leads to T cell functional exhaustion. By interacting with T lymphocytes under inflammatory conditions, DCs play a key role in shaping immune responses. To determine the surface expression profiles of immune checkpoint ligands on DC subsets in the livers of mice at 24 weeks after infection, we further investigated the immune checkpoint ligands PD-L1, CD155, and CD48 and their expressions in the cDC and pDC subsets. As shown in Figure 5, the expressions of these immune checkpoint ligands were also quite variable among the cDC and pDC subsets. The expressions of PD-L1 and CD155 were significantly higher in both the cDC and pDC subsets in the *E. multilocularis* infection group compared to the control group (Figure 5A–C). However, no significant changes in the expression of CD48 in the cDC or pDC subsets after infection were observed (Figure 5A,D). However, the expression levels of CD48 in both the cDC and the pDC subsets were higher than those of PD-L1 and CD155.

## 4. Discussion

In general, DCs are key regulators of the immune system and are widely considered professional APCs that capture, process, and present various exogenous and endogenous antigens to T lymphocytes, thereby interacting with T lymphocytes and contributing to T cell activation or tolerance in liver organs under different conditions [31]. Therefore, DCs play an important role in maintaining the unique tolerogenic environment of the liver and have been reported to be associated with persistent infection by hepatotropic pathogens, including *E. multilocularis* infections almost exclusively located in the human liver [32]. However, due to the highly heterogeneous characteristics of DCs, it is difficult to exactly modulate DC functions to effectively initiate T cell-mediated immune responses as a treatment for AE. Here, we explored the immunological characteristics of hepatic DCs in the livers of AE patients and a mouse model. We demonstrated that cDCs significantly infiltrated into the liver after *E. multilocularis* infection and exhibited a tolerogenic phenotype at the early infection stage. Immune checkpoint molecules (LAG3 and CD244) and immune checkpoint ligands (CD48 and CD86) were expressed mainly in hepatic cDC subsets in the livers of AE patients and in a mouse model. Similarly, the expression levels of PD-L1 and CD155 in cDC and pDC subsets were upregulated in the *E. multilocularis* infection group, which suggested that these cells could impair T cell function.

The functional state of DCs is critical for the induction of adaptive immunity and tolerance [33]. Accumulating evidence has demonstrated that helminthic parasites have developed multiple strategies to inhibit DC function. Previous research has reported that *Brugia malayi* microfilariae infection can induce DC apoptosis, which strongly limits the capacity of DCs to produce the proinflammatory cytokine IL-12 and prevents T cell activation and proliferation [34]. Furthermore, apoptotic DCs are rapidly taken up by immature DCs, which prevents the subsequent maturation of immature DCs in response to TLR stimuli [35]. In cestode infections, Reyes et al. reported that the maturation of DCs from susceptible mouse strains to *Taenia crassiceps* cysticercosis was impaired when the parasites were preincubated with parasite-E/S products, which caused DC tolerance [36]. However, in *E. granulosus*, a species closely related to *E. multilocularis,* researchers have found that different proteins in *E. granulosus* stimulate different activation phenotypes in DCs and that exposure of DCs to the laminated layer mucin meshwork induces a “semimature” phenotype [37]. Additionally, exposure of DCs to hydatid cyst fluid (HCF) appears to induce an immature phenotype and promote Th2-dependent secretion of cytokines, thereby inducing host immunosuppression to ensure parasite survival [38]. Furthermore, in the case of *E. multilocularis,* previous studies have demonstrated that the ES products of *E. multilocularis* can induce DC apoptosis and tolerogenic properties in vitro [24,39], and an in vivo study showed that peritoneal DCs from *E. multilocularis*-infected mice downregulated DC maturation-associated surface markers and specifically modulated CD4^+^Foxp3^+^ and CD8^+^Foxp3^+^ regulatory T cell differentiation, suggesting the potentially important role of DCs in immunosuppressive mechanisms during AE [8]. Notably, although *E. multilocularis* infections almost exclusively occur in the human liver [32], the immunological characteristics of hepatic DCs in the AE microenvironment have not been fully investigated. In this study, we used a hepatic experimental mouse model of *E. multilocularis* that closely mimics the environment at the site of infection [11] to study the dynamic changes in hepatic DC phenotypes and functions during *E. multilocularis* infection. Our data showed that the percentage of hepatic cDC subsets, which are potent APCs [40], was significantly greater in *E. multilocularis*-infected mouse livers at early and late stages, whereas the percentage of hepatic pDC subsets, a subset of DCs that have the unique ability to rapidly produce type I IFN upon viral infection [16,41], was significantly decreased. A reduction in hepatic pDCs may lead to a reduction in type I IFN production, facilitating early parasite colonization. We also investigated the expressions of CD40, CD80, and CD86 in hepatic cDC and pDC subsets to evaluate their maturation/activation status. Our data showed that early stage cDCs in the liver exhibit an immature or tolerant state characterized by low expression of the costimulatory molecules CD40 and CD80, indicating that early infection with *E. multilocularis* is a strong inducer of DC tolerance, which likely accounts for the generation of an immunosuppressive microenvironment during the infection phase in the liver, promoting successful liver colonization.

In addition, the effects of DCs on adaptive immune responses depend not only on the maturation state of DCs but also on the molecules expressed on their surface [42]. Immune checkpoint molecules play critical roles in facilitating tumor cell escape from host immune surveillance [43]. In recent years, accumulating evidence has shown that immune checkpoint molecules (LAG3, PD-1, and CTLA-4) are highly expressed in T cells and negatively regulate the function of T cells in immune responses [44,45]. Our recent research showed that the checkpoint molecules TIGIT and LAG3 are aberrantly expressed in T cells, leading to T cell exhaustion during *E. multilocularis* infection, thus accelerating disease progression [9,12].

However, some published studies have reported that immune checkpoint molecules, such as LAG3 and TIM-3, are also expressed in DCs [22,42]. Therefore, signals generated from these immune checkpoint molecules on the DC surface help to induce the tolerogenic DCs. Thus, we investigated the differential expressions of several major immune checkpoint molecules in hepatic DC subsets. We first analyzed our scRNA-Seq dataset of AE patients [27] and observed important differences in the expression of checkpoint molecules among DC subsets. Specifically, cDCs are endowed with a unique immune checkpoint repertoire characterized by high CD244, LAG3, CTLA4, and TIGIT expressions and low PD-1 expression, whereas pDCs highly express PD-1. A mouse model of *E. multilocularis* infection also confirmed that cDCs highly express CD244 and LAG3, which can help to determine whether the clinical efficacy of immune checkpoint inhibitors may rely, to some extent, on their effects on DCs.

Moreover, the interaction between DCs and T cells is dependent on receptor‒ligand interactions at various immune checkpoints, and the receptor‒ligand pathway is critically involved in immune modulation by delivering inhibitory signals to maintain the balance of T cell activation or tolerance [46,47]. When DCs express immune checkpoint ligands, such as PD-L1, they can suppress T cell immunity [43]. We further investigated the expression of the immune checkpoint ligands PD-L1, CD155, and CD48 in hepatic DC subsets in both human and murine settings and observed that cDCs and pDCs expressed different levels of these ligands; corresponding to the highly expressed receptor CD244, its ligand, CD48, also showed dominant expression in cDCs. In addition, we observed that the expressions of PD-L1 and CD155, ligands of PD-1 and TIGIT, were significantly upregulated in both the cDC and pDC subsets after *E. multilocularis* infection, which indicated that the upregulation of PD-L1 or CD155 on the surface of DCs could promote T cell functional exhaustion during the development of AE. Therefore, it can be reasonably assumed that blocking immune checkpoint interactions between DCs and T cells with ligands could increase the efficacy of DC-mediated activation of cytotoxic T cells.

In summary, this work clearly defined the immunological characteristics of DCs in the liver immune microenvironment in AE patients and a murine model. We demonstrated that DC subsets are characterized by differential expression of major costimulatory and immune checkpoint molecules as well as their ligands. The data presented here may provide novel insights into hepatic DC heterogeneity in the AE microenvironment and lay a suitable foundation for studying the role of hepatic DC subsets in AE and guiding DC targeting treatments.

## Data Availability

All of the study data are included in the article and/or Supporting Information.

## Supplementary Materials

The following supporting information can be downloaded at: https://www.mdpi.com/article/10.3390/tropicalmed9050095/s1, Figure S1: Violin plots representing the expression levels of costimulatory molecules, immune checkpoint ligands, and immune checkpoint molecules in the cDC and pDC subsets. Table S1: List of monoclonal antibodies used in this study. Data S1: The Excel spreadsheet file contains the raw data for 1, 2, 3, 4, and 5.
