# Supplementary material for: Immunological Characteristics of Hepatic Dendritic Cells in Patients and Mouse Model with Liver Echinococcus multilocularis Infection
Source: Trop Med Infect Dis. 2024 Apr 25;9(5):95. doi: 10.3390/tropicalmed9050095 (PMC11125766; doi:10.3390/tropicalmed9050095)
Supplement: Supplementary file 1 [file tropicalmed-09-00095-s001.zip › supplementary files/Table S1.docx]

**Table S1.** List of monoclonal antibodies used in this study

| **Maker** |  | **Description** | **Clone NO.** | **Conjugate** | **Manufacturer** | **Cat NO.** |
| --- | --- | --- | --- | --- | --- | --- |
| CD45 |  | Leukocyte common antigen | 30-F11 | 5.5 | biolegend | 103132 |
| CD3 |  | Lineage maker-T cells | 17A2 | FITC | biolegend | 100204 |
| CD19 |  | Lineage maker-B cells | 6D5 | FITC | biolegend | 115506 |
| NK1.1 |  | Lineage maker-NK cells | PK136 | FITC | biolegend | 108706 |
| CD317 |  | pDC maker | 927 | APC | biolegend | 127015 |
| MHC II |  | DC maker | M5/114.15.2 | BV510 | biolegend | 107635 |
| MHC II |  | DC maker | M5/114.15.2 | 594 | biolegend | 107648 |
| CD11c |  | cDC maker | N418 | PE | biolegend | 117308 |
| CD11c |  | cDC maker | N418 | PE-cy7 | biolegend | 117317 |
| LAG3 |  | Immune checkpoint molecules | C9B7W | BV421 | biolegend | 125221 |
| CD244 |  | Immune checkpoint molecules | m2B4 (B6)458.1 | 5.5 | biolegend | 133513 |
| CD244 |  | Immune checkpoint molecules | m2B4 (B6)458.1 | PE | biolegend | 133508 |
| PD-1 |  | Immune checkpoint molecules | 29F.1A12 | 594 | biolegend | 135228 |
| TIGIT |  | Immune checkpoint molecules | 1G9 | PE | biolegend | 142104 |
| CTLA-4 |  | Immune checkpoint molecules | UC10-4B9 | BV421 | biolegend | 106311 |
| CD155 |  | Checkpoint ligands | TX56 | PE | biolegend | 131508 |
| PD-L1 |  | Checkpoint ligands | 10F.9G2 | BV421 | biolegend | 124315 |
| CD48 |  | Checkpoint ligands | HM48-1 | 594 | biolegend | 103437 |
| CD40 |  | Costimulatory molecules | 3/23 | PB | biolegend | 124626 |
| CD86 |  | Costimulatory molecules | GL-1 | PE-cy7 | biolegend | 105014 |
| CD80 |  | Costimulatory molecules | 16-10A1 | BV650 | biolegend | 104731 |
