# Supplementary figures and images for: Immunological Characteristics of Hepatic Dendritic Cells in Patients and Mouse Model with Liver Echinococcus multilocularis Infection
Source: Trop Med Infect Dis. 2024 Apr 25;9(5):95. doi: 10.3390/tropicalmed9050095 (PMC11125766; doi:10.3390/tropicalmed9050095)

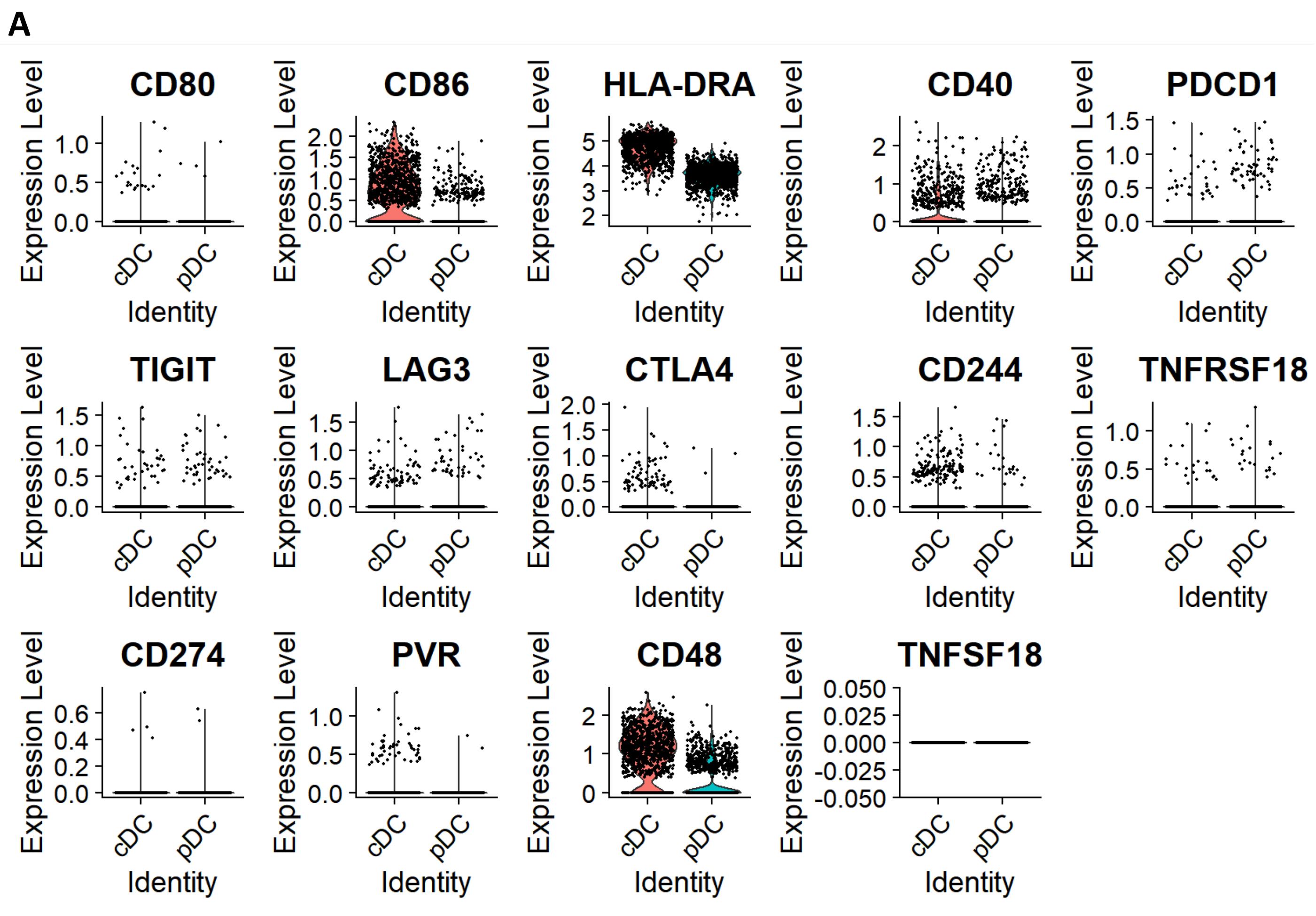

Supplement: Supplementary file 1 [file tropicalmed-09-00095-s001.zip › supplementary files/Figure S1.jpg]
